# Role of histological type on surgical outcome and survival following radical primary tumour debulking of epithelial ovarian, fallopian tube and peritoneal cancers

**DOI:** 10.1038/bjc.2011.455

**Published:** 2011-11-01

**Authors:** E-I Braicu, J Sehouli, R Richter, K Pietzner, C Denkert, C Fotopoulou

**Affiliations:** 1 European Competence Center for Ovarian Cancer Department of Gynecology, Charité, Campus-Virchow-Clinic/University-Hospital, Augustenburger Platz 1, Berlin 13353, Germany; 2 Institute of Pathology, Charité Hospital, University Medicine of Berlin, Berlin 13353, Germany

**Keywords:** ovarian carcinogenesis, type I and type II tumours, tumour pattern, survival, primary cytoreduction

## Abstract

**Background:**

To assess the clinical impact of the two histological types as designated in the proposed model for ovarian tumourigenesis in primary epithelial ovarian, fallopian tube or peritoneal cancer (EOC) patients.

**Methods:**

All consecutive EOC patients (*n*=632) after primary tumour debulking in our institution (09/2000–08/2010) were classified into one of two groups: type I tumours (*n*=100; 15.8%) composed of low-grade serous, low-grade endometrioid, clear cell, mucinous and transitional carcinomas; and Type II tumours (*n*=532; 84.1%) composed of high-grade serous, high-grade endometrioid, undifferentiated and malignant mixed-mesodermal tumours. Kaplan–Meier and logistic/Cox-regression analyses were performed to assess the impact of histological type on surgical outcome and survival.

**Results:**

Type II patients had a significantly higher incidence of advanced disease (FIGO III/IV) than Type I patients (79.8% *vs* 38%, respectively; *P*<0.001). Median CA125 values (438 *vs* 93 U ml^−1^; *P*=0.001); operative time (258 *vs* 237 min; *P*=0.001); and incidence of incomplete tumour resection (34.4% *vs* 15% *P*<0.001) were significantly higher in patients with Type II. During a mean follow-up time of 23 months (range: 1–106), 17% of patients with type I *vs* 34.8% of patients with type II tumours relapsed and/or died (*P*<0.001). Overall survival (*P*=0.021) and progression-free survival (*P*=0.003) were also significantly higher in patients with type I tumours. Multivariate analysis, while identifying postoperative tumour residuals, positive lymph nodes and extrapelvic dissemination as independent predictors of survival, failed to demonstrate any prognostic significance of histological type.

**Conclusion:**

Type I EOC patients appear to present at earlier stages have significantly higher survival and more optimal surgical outcome compared with type II patients. However, in advanced stages, histology loses significance as an independent prognosticator.

In the beginning of this century a novel tumour progression and origination model for ovarian carcinoma was proposed based on morphological and molecular genetic analyses. In this model, epithelial ovarian tumours are divided into two categories designated type I and type II, which correspond to the two main pathways of tumourigenesis (Singer *et al*, 2002; Shih and Kurman, 2004). Differences between the two types of tumours are based on clinical behaviour and evolution, genetic stability and molecular genetic profiles. As first suggested by Shih and Kurman (2004), type I tumours generally behave in an indolent manner, are genetically stable without the classic mutations such as of TP53 and tend to be confined to the ovary. On the contrary, type II tumours are characterised by a more ‘aggressive’ behaviour, the vast majority display TP53 mutations and are distinguished by rapid evolution (Shih and Kurman, 2005; Kurman and Shih, 2008, 2010). As a result of this hypothesis a 2-tier, as opposed to the 3-tier, system has been proposed, in which tumours would be subdivided into low-grade and high-grade differentiation. This approach is supposed to be more simplified, reproducible, and apparently closer to the novel biological evidence of epithelial ovarian, fallopian tube or peritoneal cancer (EOC) pathway development (Shih and Kurman, 2004; Bell, 2005; Kurman *et al*, 2008; Vang *et al*, 2009).

Even though various histopathological analyses and evaluations have been conducted to attempt to define the clinicopathological and biological features of these two tumour classifications, no analysis has yet been presented that assesses these two tumour ‘types’ relative to actual clinical outcome in a large population-based study. The present work is an attempt to assess and identify the actual clinical impact on both surgical outcome and survival of these two histological types in a large cohort of primary EOC patients who underwent optimal primary tumour debulking and first-line platinum-based chemotherapy.

## Materials and Methods

A systematic retrospective analysis of a prospectively maintained database was performed to evaluate the intraoperative tumour dissemination pattern, surgical outcome and survival of all women operated on for primary EOC in the European Competence Center for Ovarian Cancer at the Charité University of Berlin between September 2000 and August 2010. All patients who underwent a neo-adjuvant chemotherapy approach with subsequent interval debulking surgery were excluded from the present analysis (*n*=53). Patients were classified into two groups: type I and type II as introduced by Shih and Kurman in the proposed model for ovarian tumourigenesis (Shih and Kurman, 2004, 2005). Type I included all low-grade serous papillary and low-grade endometrioid, all mucinous, clear cell and transitional cell cancers. Type II included all high-grade serous papillary and high-grade endometrioid ovarian cancers, all mixed histologies and all carcinosarcomas. A total of 632 evaluable patients were included; 100 type I (15.8%) and 532 type II (84.17%).

The histopathological assessment was performed prospectively at the Institute of the Charité, all tumours were assessed by two pathologists. To control for tumour heterogeneity multiple large H&E sections of the primary ovarian lesion, lymph nodes and all intraperitoneal lesions were examined. Histological tumour typing was performed according to the WHO guidelines. Grading was performed according to Silverberg. Histological results were retrospectively regrouped as indicated by Shih and Kurman (2004). In order to detect possible misclassification, we re-examined the type I tumours with advanced FIGO stages IIIc or IV (*n*=33) and confirmed histological diagnosis.

All operations were performed by one of three gynaecologic oncologic surgeons. Staging was performed and defined in accordance with the FIGO criteria for ovarian cancer (FIGO – International Federation of Gynecology and Obstetrics, 1987). Each primary surgery was performed per midline laparotomy aiming at maximal tumour reduction and adequate staging. A summary of the surgical procedures performed is presented in detail in Table 2.

**Figure 1:**
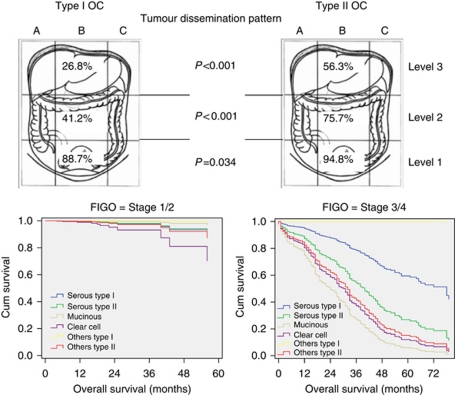
Tumour dissemination patterns in type I *vs* type II primary ovarian cancer (OC), according to the ‘Intraoperative Mapping of Ovarian Cancer’ documentation tool and survival curves according to histology depicted separately for FIGO (International Federation of Gynecology and Obstetrics) stages I/II and III/IV.

In every patient, a detailed tumour pattern was intraoperatively assessed based on the surgical procedures performed and through a systematic interview of the surgical team. Postoperatively all histological findings and associated data were entered into a validated documentation system (IMO: Intraoperative-Mapping-of-Ovarian-Cancer), specifically developed for ovarian neoplasms with particular focus on the description of the tumour pattern, maximal tumour burden, postoperative tumour residuals (0, <0.5, <1, <2, >2 cm) and the amount of preoperative ascites (none, < or >500 ml). IMO represents a detailed surgical and histopathological documentation system developed in our clinic in order to obtain a better and more objective description of the ovarian tumour spread within the abdominal cavity and to define more precisely the histopathological features of the malignancy (Sehouli *et al*, 2003, 2009, 2010a, 2010b; Fotopoulou *et al*, 2010, 2011). Within the Tumour Bank Ovarian Cancer project (www.toc.network.de), tumour tissue, ascites, serum and blood were collected from each EOC patient. The patients’ informed consent was always obtained prior to surgery and sample collection and documentation. The levels and fields according to which the abdominal cavity is divided into are presented in Figure 1.

### Follow up

All relevant patient data, including medical history, follow-up and survival data, were abstracted from the patients’ records. Survival data were last updated on 02/2011 based on the patient's files and/or responses from their physicians or insurance companies.

Patients were regularly evaluated post-treatment for evidence of disease recurrence. Clinical examinations, transvaginal and transabdominal ultrasound, serum CA125 (if preoperative value was elevated) assays were performed every 3 months. A CT/MRI scan was ordered if the above examinations revealed any pathology. Recurrence was defined by pathological, clinical, radiological or sonographical findings. Time to recurrence was defined as the date of radiological evidence of recurrence and not merely due to an isolated increase of CA125.

### Statistics

Fisher's exact test, Kendall's tau-b and Mann–Whitney's *U-*test were used for univariate analysis, where appropriate. Crude and adjusted odds ratios with corresponding 95% confidence intervals (95% CI) were obtained using logistic regression analysis. Estimates of survival were calculated using the Kaplan–Meier method, and log-rank tests were used for univariate statistical comparisons. The relative value of individual variables as independent predictors of overall survival (OS) and progression-free survival (PFS) was analysed with the multivariate Cox proportional hazard-regression model. Adjusted hazard ratios (HRs) and 95% CI for prognostic factors were estimated. All data were analysed using PASW Statistics 18 (SPSS, Chicago, IL, USA), and *P*<0.05 (two-tailed) was considered statistically significant. The follow-up time was calculated starting on the day of surgery.

## Results

A total of 632 patients were included in the present analysis. One hundred patients (15.8%) were classified as type I, whereas 532 patients (84.1%) were of type II. The distribution of low-grade serous, clear cell and mucinous carcinomas by FIGO stage was as follows: low-grade serous FIGO I: 4 (17.4%), FIGO II: 2 (8.7%), FIGO III: 17 (74%) and FIGO IV: 0; clear cell FIGO I: 8 (66.7%), FIGO II: 2 (16.7%), FIGO III: 2 (16.7%) and FIGO IV: 0; mucinous FIGO I: 19 (56%), FIGO II: 1 (2.9%), FIGO III: 8 (23.5%) and FIGO IV: 17.6% (*P*<0.001, by *χ*^2^-test). The detailed distribution of the 33 advanced type I tumours (i.e., IIIc/IV) was as follows: low-grade serous 13 (39.4%), mucinous 14 (42.4%), clear cell 2 (6.1%) and transitional cell 4 (12.1%). Patient characteristics are presented in detail in Table 1. Type I patients were significantly younger, presented at significantly earlier FIGO stages and had lower rates of positive lymph nodes, preoperative ascites and CA125 values.

During a mean follow-up time of 23 months (range: 1–106; median: 15), 202 patients (32%) relapsed and died. Estimated 5-year OS rates were 56.3% (95% CI: 38.3–84.0%) for type I patients *vs* 39.3% (95% CI: 32.6–46.0%) for type II patients (*P*=0.021) and thus statistically significantly different. Estimated 2-year PFS rates were also significantly better for type I compared with type II patients: 59.8% (95% CI: 46.1–73.4%) *vs* 44.9% (95% CI: 39.6–50.2%); *P*=0.003. Survival curves are presented in Figure 1. However, when considering only advanced FIGO IIIc/IV patients, both OS and PFS were not statistically significantly different between the two groups: median OS was 35 months (95% CI: 13.06–56.93) for type I *vs* 40 months (95% CI: 33.5–46.47) for type II patients (*P*=0.779) and median PFS was 20 months (95% CI: 0.000–42.38) for type I *vs* 17 months (95% CI: 14.49–19.5) for type II patients (*P*=0.714).

Type I patients demonstrated higher rates of platinum sensitivity after first-line platinum-based chemotherapy (85.4% *vs* 77.5%) relative to type II patients, but failed to reach any statistical significance (*P*=0.314).

Detailed surgical procedures performed during primary tumour debulking are presented in Table 2. Type I patients underwent significantly less frequent para aortic lymph node dissection, large bowel resection, an extensive peritonectomy and a diaphragmatic resection, and had a significantly lower overall complication rate during a significantly less median operative time. These differences lost statistical significance when comparing only the FIGO stages III and IV patients. Of the 33 type I patients with advanced disease, the vast majority (84%) underwent an optimal tumour debulking; i.e., 72% were surgically completely tumour-free and 12% had tumour residuals of less than 0.5 cm. These rates were equivalent to the optimal tumour resection rates of type I patients across all stages.

Tumour dissemination rates were significantly lower in all three abdominal levels (i.e., IMO 1–3) in type I patients. Exact values are presented in Figure 1. In comparison with type II patients, type I patients had lower rates of tumour involvement of the omentum (33% *vs* 66% *P*<0.001), the Pouch of Douglas (11% *vs* 23% *P*=0.007), the pelvic peritoneum (17% *vs* 30% *P*=0.007), the diaphragm (19% *vs* 42% *P*<0.001), the serosa of the small (15% *vs* 31% *P*=0.001) and large (24% *vs* 51% *P*<0.001) intestine, the mesentery (20% *vs* 41% *P*<0.001), as well as significantly lower rates of diffuse peritoneal carcinosis (42% *vs* 78% *P*<0.001). Although the mean number of extracted lymph nodes was not significantly different between the two patient groups (28 for type I *vs* 31 for type II; *P*=0.25), the mean number of affected lymph nodes was significantly higher in type II patients (5.8 *vs* 1.5; *P*=0.001).

The mean number of IMO fields with tumour residuals was significantly higher in type II *vs* type I patients (0.45 *vs* 0.87; *P*<0.001); the number of IMO fields with maximal tumour load was also significantly greater in type II patients (mean: 1.1 *vs* 1.4; *P*<0.001), as was the total number of IMO fields affected with tumour (mean: 2.7 *vs* 4.3; *P*<0.001).

On multivariate analysis, comparing the different histological entities stage by stage, only a positive lymph node status was identified as independent predictor negatively affecting OS in early tumour stages 1 and 2, while absence of ascites had a significant protective effect. For the more advanced tumour stages 3 and 4, positive lymph node status as well as any postoperative tumour residuals and mucinous histology appeared to negatively affect survival. These data are presented in Table 3.

Interestingly, when evaluating only the tumour-free-operated patients, high-grade serous cancers (HR: 3.83; 95% CI: 1.062–13.8; *P*=0.04), all the other type II histologies (HR: 4.89; 95% CI: 1.18–20.14; *P*=0.03) and multifocal tumour dissemination (HR: 1.8; 95% CI: 1.08–2.9; *P*=0.023) were the only independent predictors negatively affecting survival. Ascites, lymph node status and age did not seem to have any significant effect.

Independent predictors of tumour recurrence were for the early stages 1 and 2, a positive lymph node status, while absence of ascites appeared to have a significant protective effect. For the more advanced tumour stages 3 and 4, a positive lymph node status and the presence of tumour residuals were of prognostic significance for relapse. Data are presented in Table 3.

Predictors of complete tumour resection and risk factors for extrapelvic tumour dissemination are presented in Tables 4 and 5.

Regarding operative morbidity, only intestinal resection was shown to be an independent risk factor, between the histological types, advanced age, ascites, FIGO stage, lymph node dissection, operative time and tumour residuals.

## Discussion

In the present analysis we evaluated the impact of histological type, as defined by the dualistic model of carcinogenesis, on surgical and clinical outcome after primary tumour debulking of EOC patients. We demonstrated that type I patients were significantly younger at initial presentation of disease compared with patients with type II tumours. Moreover, in type I patients the disease presented at significantly earlier stages, with lower incidence of ascites and lymph node involvement, significantly higher rates of optimal tumour debulking and, consequently, significantly better overall and PFS rates. Nevertheless, when considering only the subgroup of patients with advanced FIGO stages III and IV, the histological type did not retain any significant prognostic value on survival nor on surgical outcome. Furthermore, we could not identify any significant independent effect of the different histological entities on tumour respectability or operative morbidity. In advanced stages of disease, only mucinous histology had a negative impact on survival when compared with low-grade serous tumours, whereas high-grade serous histology was identified as a significant risk factor for extrapelvic tumour dissemination.

Interestingly, in the tumour-free-operated patients, type II cancers were together with a multifocal tumour dissemination, the only significant risk factor negatively affecting OS.

This is, to our knowledge, the first report of assessing the impact and significance of tumour histology using a validated and systematic documentation tool such as IMO, where intraoperative tumour dissemination, operative procedures and tumour residuals are described and recorded in a prospective and systematic manner using specifically designed schemes and figures (see Figure 1), avoiding potential bias and errors in the assessment of tumour dissemination and site of tumour residuals. In previous studies, Gershenson *et al* (2009) investigated the chemoresistance of recurrent low-grade serous ovarian carcinoma compared with high-grade ovarian cancers. The authors questioned whether the latter's high rate of stable disease was due more to the tumour's biology or to the influence of chemotherapy.

Even though the existing guidelines regarding EOC do not officially include the histological subtype in the decision-making process, there is increasing evidence that indicates histology has a significant role in the overall patients’ outcome and prognosis (Goff *et al*, 1996; Ho *et al*, 2004; Pectasides *et al*, 2005; Bamias *et al*, 2010; Wimberger *et al*, 2010; Zaino *et al*, 2010), while multiple papers describe treating low-grade serous, mucinous and clear cell cancers differently. The Gynecologic Oncology Group (GOG) has designed several large multicentre phase III trials, specifically for low-grade serous cancers alone, mucinous alone and clear cell alone, attempting to treat, for instance, mucinous type tumours similar to intestinal cancers with Oxaliplatin and Capecitabine ± Bevacizumab.

Epithelial ovarian, fallopian tube or peritoneal cancer is complex at both the histopathological and molecular level, being composed of several histological subtypes that are, in of themselves, heterogeneous and contain distinct molecular signatures (Blagden and Gabra, 2009). It appears that rare histological subtypes, including mucinous or clear cell adenocarcinomas, respond rather poorly to the standard first-line chemotherapy combination of carboplatin and paclitaxel, and are associated with a more dismal outcome compared with their serous counterparts (Blagden and Gabra, 2008). This issue was also addressed in a previous analysis by Goff *et al* (1996), where 70% of the patients with clear cell histology developed progressive disease, compared with only 29% of those with serous histology. Similar lower response rates to first-line platinum-based chemotherapy were also shown for patients with mucinous epithelial ovarian cancer (Pectasides *et al*, 2005; Bamias *et al*, 2010). In a recent retrospective analysis of the German Arbeitsgemeinschaft Gynaekologische Onkologie on a large cohort of EOC patients (Wimberger *et al*, 2010), multivariable analysis for OS identified mucinous histological type next to postoperative residual tumour, multiple sites of metastases and ECOG performance status as statistically significant prognostic variables.

In a further study by the GOG (Zaino *et al*, 2010), advanced stage mucinous adenocarcinoma of the ovary is being characterised as highly lethal with highly significantly lower OS rates compared with women with serous carcinoma (14 *vs* 42 months; *P*<0.001).

Interestingly, according to the ovarian tumourigenesis hypothesis, both clear cell and mucinous adenocarcinomas belong to the prognostically more favourable type II group. In our analysis we demonstrated that both entities are indeed associated with a more favourable prognosis, mainly due, however, to the fact that they initially present at earlier stages. In advanced stages of the disease, histological type I *vs* type II cancers did not show significant differences with respect to survival or surgical outcome, and advanced type I cancers behaved as aggressively as type II cancers, with mucinous histology even being associated with a significantly worse outcome compared with the serous type. How might we explain this? One theory is that it is a reflection of the p53 status. It is known that mutations in p53 are common in high-grade serous carcinomas in contrast to their low-grade serous counterparts in which mutations in p53 are rather rare. Many studies have shown that 50–80% of advanced stage disease harbours mutant p53, presumably because they might possibly originate from high-grade serous carcinomas (Shih and Kurman, 2004). In an immunohistochemical evaluation by Eltabbakha *et al* (2004) aiming to investigate the clinical and molecular factors associated with cytoreduction among women with advanced stage epithelial EOC, it was found that p53 expression was a highly significant predictor for cytoreducibility. Women whose tumours showed mild or moderate p53 expression were 5.6 times more likely to achieve complete cytoreduction compared with women whose tumours showed strong p53 expression (Eltabbakha *et al*, 2004). Projecting this to the well-established fact that in advanced stages, tumour residual disease is the most significant prognostic factor for survival and that optimal tumour debulking to microscopic residuals is regarded as the cornerstone of therapeutic management in EOC, one could surmise that p53 status has a significant impact on overall prognosis even in advanced stage disease. In our analysis we did not perform any p53 mutation analysis or an assessment of the Ras/Raf wild type, which should be noted as shortcoming of this study. We however, performed a subanalysis of the prognostically more favourable cohort of the tumour-free-operated patients and showed that type II histology was a negative prognosticator of survival. This might be hence a sign of a potential ‘higher aggressiveness’ of type II cancers after all, whereas the exact underlying mechanisms have to be investigated in future trials.

A further weakness of the present analysis is the relatively short follow-up of an average of 23 months.

It is known that underlying the general high mortality of EOC is the molecular behaviour of the disease, with ∼75% of patients presenting at an advanced clinical stage, in terms of a high-volume disease with dissemination in the entire abdominal cavity (Kurman *et al*, 2008; Blagden and Gabra, 2009). Type I tumours have been described as slow growing, as evidenced by the observation that they are large and often confined to the ovary at diagnosis (Shih and Kurman, 2004). This observation was corroborated in this study, where type I tumours initially presented in approximately half of the cases at an early stage restricted to the pelvis (IMO level 1), as opposed to only 14% of type II patients. This may imply that despite the fact that rare entities such as mucinous and clear cell carcinomas show a poorer response to conventional chemotherapeutic regimes, their tendency to be diagnosed at earlier stages, confined to the pelvis, may actually result in a more favourable prognosis and more beneficial profile of the type I tumours.

However, these hypotheses have to be verified in prospective morphological and molecular genetic studies, where a molecular biological profiling of the tumour will be conducted at inception of the disease and, subsequently, correlated with surgical outcome and survival. In this way a new rationale in the approach to detection, therapeutic management and follow-up may be developed, which would be closer to the actual tumour biology profiling and large-scale heterogeneity of the disease (Gershenson, 2010) and may allow a more individualised and potentially more effective management.

## Figures and Tables

**Table 1:** Demographic, tumour-related and operative characteristics of the entire patient cohort and further classified according to type I *vs* type II ovarian malignant disease

	**Type I, *N*=100 (%)**	**Type II, *N*=532 (%)**	**All patients, *N*=632 (%)**	***P*-value**
Median age at primary diagnosis (years)	52 (15–86)	59 (21–92)	58 (15–92)	**<0.001**
				
*FIGO stage*				**<0.001**
I	39 (39)	47 (9.2)	86 (14.3)	
II	5 (5)	35 (6.8)	40 (6.7)	
IIIa	2 (2)	7 (1.3)	355 (59.1)	
IIIb	3 (3)	19 (3.5)		
IIIc	27 (27)	297 (55.8)		
IV	6 (6)	85 (16.6)	91 (15.1)	
				
*Grading*				**n.a.**
G1	53 (53)	3 (0.6)	56 (9)	
G2	20 (21.5)	174 (32.9)	194 (31.2)	
G3	17 (18.3)	350 (66.2)	367 (59)	
				
*Histology*				**n.a.**
Serous papillary	31 (31)	467 (87.8)	498 (78.8)	
Clear cell	15 (15)	0	15 (2.4)	
Mucinous	36 (36)	0	36 (5.7)	
Mixed/undifferentiated	0	26 (4.9)	26 (4.1)	
Transitional cell	10 (10)	0	10 (1.6)	
Carcinosarcoma	0	6 (1.1)	6 (0.9)	
Endometrioid	8 (8)	33 (6.2)	41 (6.5)	
				
*N status*				**<0.001**
N0	40 (41.2)	187 (35.3)		
N1	28 (28)	260 (49.1)		
Nx	29 (29)	83 (15.7)		
				
Distant metastases at primary diagnosis	6 (6)	93 (20)		**0.009**
				
*Intraoperative ascites*				**<0.001**
None	47 (47)	151 (28.7)	198 (31.8)	
<500 ml	33 (33)	207 (39.3)	240 (38.5)	
>500 ml	16 (16)	169 (32.1)	185 (29.7)	
				
Median preoperative CA125 (U ml^−1^)	93 (8–14 315)	438 (6–41 500)	323 (6–41 500)	**0.001**
				
Median operative time (minutes)	237 (34–457)	258 (38–592)	251 (34–592)	**0.001**
				
*Postoperative tumour residuals*				**<0.001**
None	85 (85)	342 (65.6)	427 (69.2)	
<0.5 cm	6 (6)	71 (13.6)	77 (12.5)	
0.5–1 cm	2 (2)	57 (10.9)	59 (9.6)	
1–2 cm	0	12 (2.3)	12 (1.9)	
>2 cm	3 (3)	39 (7.5)	42 (6.8)	

Abbreviation: FIGO=International Federation of Gynecology and Obstetrics. Bold values are statistically significant, i.e. *P*<0.05.

**Table 2:** Surgical procedures performed in type I and type II primary ovarian cancer patients and the associated operative morbidity

**Procedure performed**	**Type I, *N*=100 (%)**	**Type II, *N*=532 (%)**	**All patients, *N*=632 (%)**	***P*-value**
Hysterectomy	63 (63)	388 (72.9)	451 (71.45)	0.053
Pelvic lymph node dissection	65 (65)	385 (72.4)	450 (71.2)	0.149
Para aortic lymph node dissection	63 (63)	400 (75.3)	463 (73.4)	**0.013**
Partial resection urinary bladder	0	10 (1.9)	10 (1.6)	0.376
Preternatural anus	4 (4)	31 (5.8)	35 (5.6)	0.634
				
*Intestinal resection*	28 (28)	228 (42.9)	256 (40.5)	**0.005**
Small intestine	11 (11)	95 (18)	106 (16.9)	0.108
Large intestine	23 (23)	209 (39.6)	232 (36.9)	**0.002**
				
Appendectomy	55 (55)	247 (46.3)	302 (47.7)	0.127
Omentectomy	87 (87)	494 (93)	581 (92.1)	0.066
				
*Upper abdominal procedures*				
Partial liver resection	2 (2)	10 (1.9)	12 (1.9)	1.0
Liver capsule resection	7 (7)	27 (5.1)	34 (5.4)	0.46
Partial gastrectomy	0	12 (2.3)	12 (1.9)	0.23
Cholecystectomy	1 (1)	26 (4.9)	27 (4.3)	0.103
Splenectomy	2 (2)	30 (5.6)	32 (5.1)	0.208
Peritonectomy	49 (49)	368 (69.4)	417 (66.3)	**<0.001**
Diaphragmatic resection	7 (7)	80 (15.1)	87 (13.8)	**0.038**
				
*Operative complications*	15 (19)	146 (33.5)	161 (31.3)	**0.012**
Thromboembolism	2 (2)	21 (4.8)	23 (4.5)	0.555
Infection/sepsis	6 (6)	38 (8.8)	44 (8.6)	0.831
Intestinal complications (anastomotic insufficiency, fistula)	2 (2)	30 (6.95)	32 (6.3)	0.6
Organ failure	0	13 (3)	13 (2.5)	0.235
Postoperative bleeding	2 (2)	14 (3.2)	16 (3.1)	1.0
Postoperative neurological impairment	1 (1)	24 (5.5)	25 (4.9)	0.153
Pulmonary complications	3 (3.8)	37 (8.5)	40 (7.8)	0.176
Death within 30 days	1 (1.2)	11 (2.3)	12 (2.2)	1.0
Relaparotomy	3 (3.8)	25 (5.8)	28 (5.5)	0.6

Bold values are statistically significant, i.e. *P*<0.05.

**Table 3:** Risk factors for mortality and ovarian cancer recurrence – multivariate analysis in type I *vs* type II ovarian cancer patients

	**In tumour stages 1 and 2**			**In tumour stages 3 and 4**		
**Variable**	**HR**	**95% CI**	***P*-value**	**HR**	**95% CI**	***P*-value**
*Mortality*						
No ascites^a^ (*vs* any)	0.33	0.002–0.48	**0.013**	1.07	0.661–1.72	0.79
Ascites <500 ml^a^ (*vs* >500 ml)	0.036	0.004–0.331	**0.003**	0.87	0.61–1.24	0.46
N1 (*vs* N0)	110.87	6.78–1812	**0.001**	2.63	1.73–4	**<0.001**
Any tumour residuals (*vs* none)	11.64	0.57–238.04	0.11	1.95	1.35–2.8	**<0.001**
High-grade serous (*vs* low-grade serous)	0.88	0.033–23.87	0.94	2.5	0.6–10.6	0.17
Mucinous (*vs* low-grade serous)	0.28	0.004–20.84	0.56	5.4	1.12–26.06	**0.03**
Clear cell (*vs* low-grade serous)	3.09	0.14–68.8	0.476	4.07	0.34–48.2	0.27
Age >65 years (*vs* <65 years)	0.14	0.002–11.95	0.38	1.4	0.98–2.001	0.06
>4 IMO fields tumour involved (*vs* <4)	2.02	0.065–62.53	0.69	1.42	0.99–2.03	0.052
Other types I (*vs* low-grade serous)	0.000	0.000–	0.99	0.00	0.000–	0.95
Other types II (*vs* low-grade serous)	1.206	0.053–27.57	0.91	3.62	0.8–16.4	0.09
						
*Ovarian cancer relapse*						
No ascites (*vs* any)	0.15	0.03–0.81	**0.028**	1.2	0.82–1.8	0.32
Ascites <500 ml (*vs* >500 ml)	0.12	0.02–0.67	**0.015**	0.86	0.63–1.17	0.33
N1 (*vs* N0)	11.37	2.02–64.1	**0.006**	1.88	1.32–2.69	**0.001**
Any tumour residuals (*vs* none)	3.96	0.43–35.92	0.221	1.66	1.2–2.27	**0.002**
High-grade serous (*vs* low-grade serous)	1.59	0.12–20.76	0.22	2.34	0.84–6.53	0.10
Mucinous (*vs* low-grade serous)	0.29	0.015–6.02	0.43	2.54	0.75–8.6	0.13
Clear cell (*vs* low-grade serous)	4.83	0.31–75.1	0.26	4.6	0.79–27.12	0.09
Age >65 years (*vs* <65 years)	0.53	0.082–3.48	0.51	1.11	0.81–1.5	0.49
Other types I (*vs* low-grade serous)	0.000	0.000–	0.99	0.61	0.066–5.6	0.66
Other types II (*vs* low-grade serous)	4.64	0.46–47.07	0.19	2.06	0.68–6.3	0.2

Abbreviations: CI=confidence interval; HR=hazard ratio; IMO=Intraoperative Mapping of Ovarian Cancer.

Variables are compared stage-by-stage (1/2 and 3/4) between the different histologies.

a Ascites with significant protective effect. Bold values are statistically significant, i.e. *P*<0.05.

**Table 4:** Predictors of complete tumour resection – multivariate analysis in type I *vs* type II ovarian cancer patients

**Variable**	**Odds ratio**	**95% Confidence interval**	***P*-value**
No ascites^a^ (*vs* any)	2.5	1.32–4.8	**0.005**
Ascites <500 ml^a^ (*vs* >500 ml)	1.98	1.2–3.3	**0.008**
N1 (*vs* N0)	0.53	0.3–0.93	**0.027**
High-grade serous (*vs* low-grade serous)	0.32	0.09–1.14	0.081
Mucinous (*vs* low-grade serous)	0.57	0.098–3.28	0.527
Clear cell (*vs* low-grade serous)	1	0.000–	0.99
Age >65 year (*vs* <65 years)	0.56	0.34–0.92	**0.021**
>4 IMO fields tumour involved (*vs* <4)	0.57	0.36–0.23	**<0.001**
Tumour upper abdomen (i.e., level 2/3; yes *vs* no)	0.36	0.005–0.331	**0.003**
Other types I (*vs* low-grade serous)	0.58	0.062–5.43	0.63
Other types II (*vs* low-grade serous)	0.25	0.06–1.08	0.063

Abbreviation: IMO=Intraoperative Mapping of Ovarian Cancer.

Variables are compared between the different histologies.

a Ascites with significant protective effect. Bold values are statistically significant, i.e. *P*<0.05.

**Table 5:** Risk factors for extrapelvic tumour dissemination (i.e., IMO levels 2+3) – multivariate analysis in type I *vs* type II ovarian cancer patients

**Variable**	**Odds ratio**	**95% Confidence interval**	***P*-value**
*All FIGO stages*			
No ascites^a^ (*vs* any)	0.078	0.03–0.205	**<0.001**
Ascites <500 ml^a^ (*vs* >500 ml)	0.156	0.06–0.40	**<0.001**
N1 (*vs* N0)	1.49	0.83–2.66	0.18
High-grade serous (*vs* low grade serous)	3.5	1.4–8.7	**0.007**
Mucinous (*vs* low-grade serous)	1.23	0.34–4.43	0.74
Clear cell^a^ (*vs* low-grade serous)	0.46	0.07–3.1	0.43
Age >65 years (*vs* <65 years)	1.07	0.60–1.9	0.82
FIGO stage 1/2^a^ (*vs* 3/4)	0.081	0.044–0.152	<0.001
Other types I^a^ (*vs* low-grade serous)	0.37	0.072–1.87	0.23
Other types II (*vs* low-grade serous)	1.14	0.39–3.34	0.8
			
*Only FIGO stages III and IV*			
No ascites^a^ (*vs* any)	0.062	0.017–0.22	**<0.001**
Ascites <500 ml^a^ (*vs* >500 ml)	0.14	0.04–0.5	**0.002**
N1 (*vs* N0)	1.06	0.54–2.10	0.85
Low-grade serous (*vs* high-grade serous)	0.33	0.10–1.04	0.06
Mucinous (*vs* high-grade serous)	0.46	0.085–2.5	0.37
Clear cell^a^ (*vs* high-grade serous)	0.27	0.014–5.4	0.39
Age >65 years (*vs* <65 years)	1.65	0.78–3.5	0.19
Other types I^a^ (*vs* high-grade serous)	0.24	0.018–3.37	0.29
Other types II (*vs* high-grade serous)	0.37	0.15–0.9	**0.03**

Abbreviations: FIGO=International Federation of Gynecology and Obstetrics; IMO=Intraoperative Mapping of Ovarian Cancer.

Variables are compared between the different histologies and separately for FIGO stages III and IV.

a Ascites with significant protective effect. Bold values are statistically significant, i.e. *P*<0.05.
